# Assessment of Chromatin Maturity in Human Spermatozoa: Useful Aniline Blue Assay for Routine Diagnosis of Male Infertility

**DOI:** 10.1155/2013/578631

**Published:** 2013-10-03

**Authors:** Afifa Sellami, Nozha Chakroun, Soumaya Ben Zarrouk, Hanen Sellami, Sahbi Kebaili, Tarek Rebai, Leila Keskes

**Affiliations:** ^1^Histology-Embryology-Biology of Reproduction Laboratory, Medical School, Sfax 3029, Tunisia; ^2^Histology Embryology Research Unit, Faculty of Medicine, Medical School, Sfax 3029, Tunisia; ^3^Gynaecology and Obstetrics Department, Hedi Chaker Academic Hospital, Sfax 3029, Tunisia

## Abstract

During spermatogenesis, sperm chromatin undergoes structural changes and results in a high condensation. This nuclear compaction would be useful as a predictor of sperm fertilization capacity and pregnancy outcome. We purpose to evaluate firstly the relationship among chromatin maturity assessed by aniline blue staining (AB) and the semen parameters in infertile men. Secondly, we analyzed whether the sperm gradient density centrifugation is effective to select mature spermatozoa. Fifty-one ejaculates were investigated by semen analysis and stained for chromatin condensation with AB to distinguish between unstained mature sperm and stained immature sperm. AB was applied also on 12 ejaculates which proceeded by density gradient centrifugation to compare the rates of immature sperm before and after selection. Neat semen were divided into two groups: G1 (*n* = 31): immature sperm <20% and G2 (*n* = 20): immature sperm ≥20%. No significant differences were detected in sperm concentration, motility, and normal morphology between G1 and G2. However, the rates of some morphology abnormalities were higher in G2: head abnormalities (*P* = 0.01) and microcephalic sperm (*P* = 0.02). We founded significant correlation between sperm immaturity and acrosome abnormalities (*r* = 0.292; *P* = 0.03). Sperm selection has significantly reduced the rates of immature sperm. A better understanding of chromatin structure and its impact on the sperm potential is needed to explore male infertility.

## 1. Introduction

Routine semen analysis is the basic analysis in the exploration of male infertility. It provides useful data concerning sperm count, sperm motility and viability, sperm morphology, performance of genital glands, and ejaculation. Although, the contribution of the semen analysis is limited on the assessment of some important criteria implicated in the sperm functional potential. In fact, it is well established that the maturity of sperm chromatin is essential for fertilizing capacity of spermatozoa and embryonic development [1–3]. The proportion of sperm with abnormal chromatin condensation in the ejaculate could be a prognostic factor in assessing the chances of fertilization and pregnancy [4]. Indeed, during spermiogenesis, sperm nuclear is completely reorganized and undergoes characteristic rearrangements with an important compaction [4–6]. This chromatin condensation involves a replacement of somatic and testis—specific nucleoproteins: histones by transition proteins then by more basic proteins named protamines P1 and P2 [5, 7] with a proper ratio of protamine 1 to protamine 2 (P1/P2) [8]. These protamines pack nuclear DNA tightly into highly condensed chromatin; in fact, the sperm nucleus will have an important mechanical and chemical stability [9]. The nuclear compaction is involved in the protection of paternal genome during the transit of spermatozoa through the male and female genital tracts and during its interaction with oocyte. Abnormalities in chromatin condensation can cause nuclear damages as DNA denaturation or fragmentation often associated with male infertility [10].

The addition of the evaluation of sperm head maturity to routine semen analysis improves the assessment of fertility in men [11]. Many analytical techniques have been proposed to assess nuclear compaction in ejaculated sperm. Some cytochemical or fluorescent dyes were used: acidic aniline blue to detect excessive presence of histones [11–15], chromomycin A3 applied for the evaluation of protamine deficiency [16, 17], and acridine orange [17] and toluidine blue staining used for assessing sperm chromatin structure and packaging [7, 18]. These different dyes have the advantage of providing suitable slides for use on a light or fluorescent microscope. Aniline blue (AB) test evaluates the degree of sperm chromatin compaction or maturation, and it is able to detect sperm chromatin defects related to their nucleoprotein content [11, 14]. This method allows discriminating between the presence of lysine-rich histones and arginine- and cysteine-rich protamines in sperm nuclei [11, 19]. Consequently, histone-rich nuclei of immature sperm will take up the blue stain whereas mature protamine-rich nuclei remain unstained. Several questions obviously arise including (i) can the (AB) test predict the male prognostic fertility? and (ii) can it be a useful complement assay to direct the treatment of couple infertility?

Many studies have shown negative correlations between the defects of sperm chromatin integrity and male fertility potential [20]. However, there is a controversy on the correlation between sperm nucleus maturity evaluated with AB staining and some semen parameters firstly and sperm fertilization capacity and embryo quality secondly. Other reports have found a negative predictive value of nuclear compaction in pregnancy outcome during assisted reproduction treatment [10]. So, the aim of sperm selection techniques applied in assisted procreation techniques is not only based on the assessment of classic parameters as sperm motility, numeration, and morphology but also involves more other sperm characteristics such as nuclear integrity.

The aims of the present study were to assess the sperm chromatin maturity in infertile men using acidic aniline blue staining and to evaluate the relationship among chromatin sperm status and the semen parameters in male infertility. Moreover, we analyzed whether the sperm gradient density centrifugation technique is effective to select mature spermatozoa.

## 2. Materials and Methods

### 2.1. Patients

Our study was carried out in 51 semen samples from male partner of infertile couple attending the Histology-Embryology Laboratory of Sfax Medical School (Tunisia) for semen investigations. The patients were aged between 28 and 49 years old with a mean age (±standard deviation (SD)) of 35.28 ± 0.62 years.

### 2.2. Collection of Semen Samples

Semen samples were collected by masturbation after 3–5 days of sexual abstinence and allowed to liquefy for 30 minutes at 37°C. 

### 2.3. Semen Analysis

Basic semen analysis consisted in the measurement of semen volume, sperm concentration (hemocytometer method), motility (total motility, rapid progressive (type a), slow progressive (type b), and nonprogressive (type c)), vitality, and morphology. All parameters were carried out according to the World Health Organization (WHO) guidelines [21]. 

### 2.4. Semen Preparation

Among these 51 samples, 12 were proceeded by Sill select density gradient centrifugation (*n* = 12). Sill select (Fertipro NV, Belgium) is a silane-coated silica particles solution that allows for separation of spermatozoa according to their density. A 2-layer gradient was prepared using, respectively, solutions of 90% and 45% Sill select. By using a sterile pipette, 1 mL of liquefied semen sample was placed on top of the upper layer in a conical 15 mL centrifuge tube. The tube was centrifuged at 300 g for 20 minutes. The supernatant was then removed and the sperm pellet was suspended in a volume of 3 mL of Ferticult medium (Fertipro NV, Belgium). It was again centrifuged at 500 g for 10 minutes. The final sperm pellet was suspended in 0.3 mL of Ferticult medium and examined for the sperm concentration, motility, morphology, and AB staining.

### 2.5. Acidic Aniline Blue Staining

For each neat semen sample (*n* = 51), we evaluated the sperm nuclei chromatin condensation using AB staining. Among these samples, we also evaluated by AB staining the nuclear maturity of spermatozoa recovered after a Sill select density gradient centrifugation (*n* = 12).

AB staining was performed as previously described by Hofmann and Hilscher [19]. After liquefaction, 1 mL of neat semen was washed twice in 0.2 M phosphate buffered (pH = 7.2). We spread 20 *μ*L of sperm pellet on glass slides, and sperm smears are allowed to dry in air. For the 12 samples on which we performed a Sill select gradient density, smears were made with 20 *μ*L from the final sperm pellet after a double washing in 0.2 M phosphate buffered (pH = 7.2). 

Smears were fixed with a solution of 3% buffered glutaraldehyde in 0.2 M phosphate buffer (pH = 7.2) for 30 minutes. Slides were then stained with 5% aqueous aniline blue solution mixed with 4% acetic acid (pH = 3.5) for 5 minutes. 

**Figure 1:**
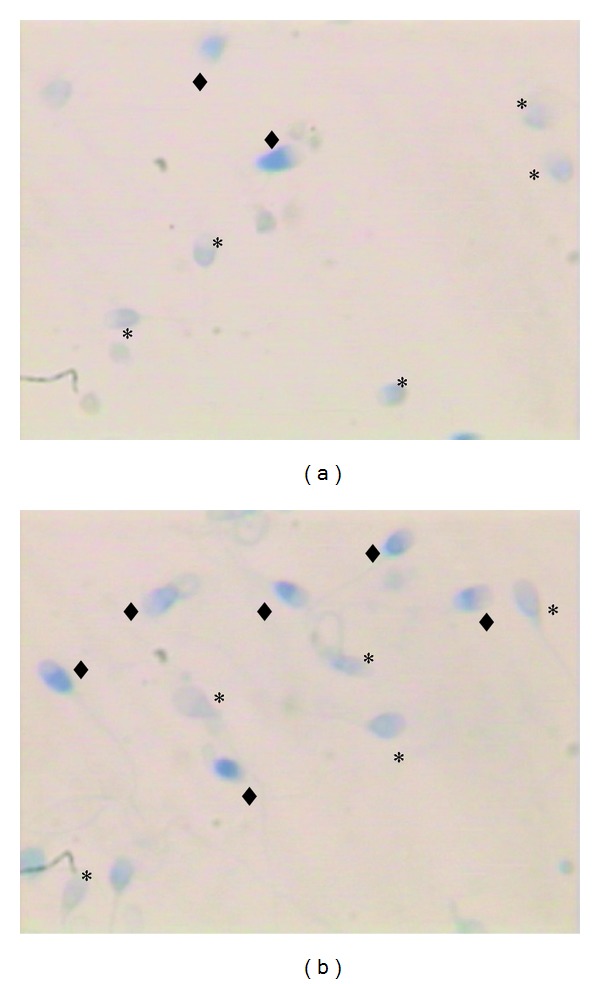
Sperm chromatin condensation assessed by aniline blue. (a) Sample showing mainly mature sperm with unstained nucleus (∗). (b) Sample showing mainly immature sperm (*◆*) with blue-stained nucleus.

For each stained smear, 200 spermatozoa were evaluated with light microscope in oil immersion magnification (100x objective). Spermatozoa with unstained nuclei are considered normal (mature chromatin) while those blue stained were considered abnormal (immature chromatin) (Figure 1). The results were expressed as percentages of nuclear unstained and stained sperm. An ejaculate with a rate of blue-stained nucleus sperm less than 20% was considered normal [4, 11].

### 2.6. Statistical Analysis

A statistical analysis was performed using SPSS 13.0 software. Statistical tests including Student's *t*-test, Pearson's and Spearman's correlations, and linear regression were used. The statistical significance was considered for *P* values <0.05.

## 3. Results

The mean values (±SD) and ranges of semen parameters, unstained (mature) sperm head (Figure 1(a)), and blue-stained (immature) sperm head (Figure 1(b)) are summarized in Table 1.

Our samples were divided into two groups: group G1 (*n* = 31): blue-stained nuclei (immature sperm) <20% and group G2 (*n* = 20): blue-stained nuclei (immature sperm) ≥20%. We compared the means values of semen parameters in each group, and we have not found any significant differences between G1 and G2 (Table 2).

The comparison of spermocytogram data showed many differences between G1 and G2 groups. The average numbers of morphological abnormalities (/100 spermatozoa analyzed) were higher in G2 concerning abnormalities of sperm head (*P* = 0.01) mainly microcephalic spermatozoa (*P* = 0.02) and acrosome abnormalities (*P* = 0.09) (Table 2).

**Figure 2:**
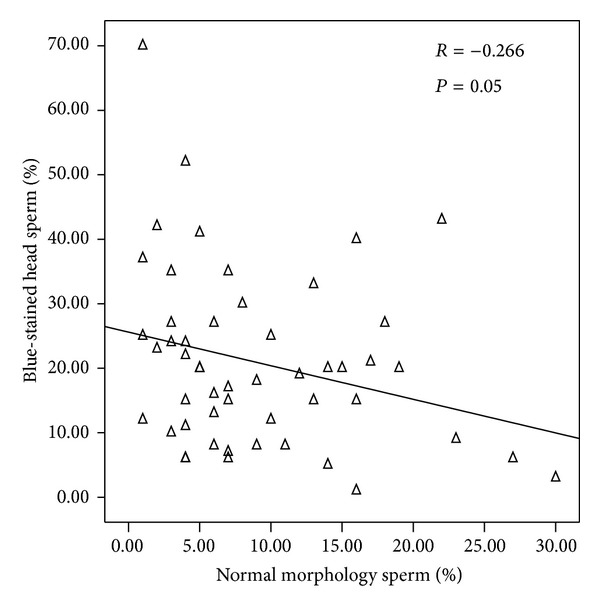
Correlation between sperm nucleus maturity and sperm morphology.

**Figure 3:**
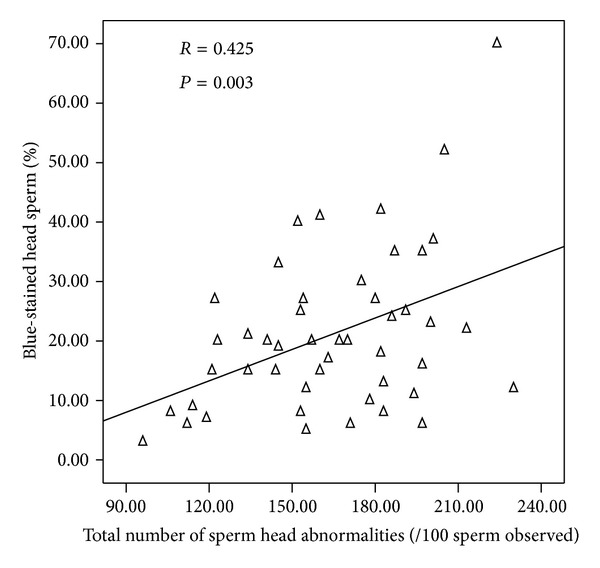
Correlation between sperm nucleus maturity and sperm head abnormalities.

**Figure 4:**
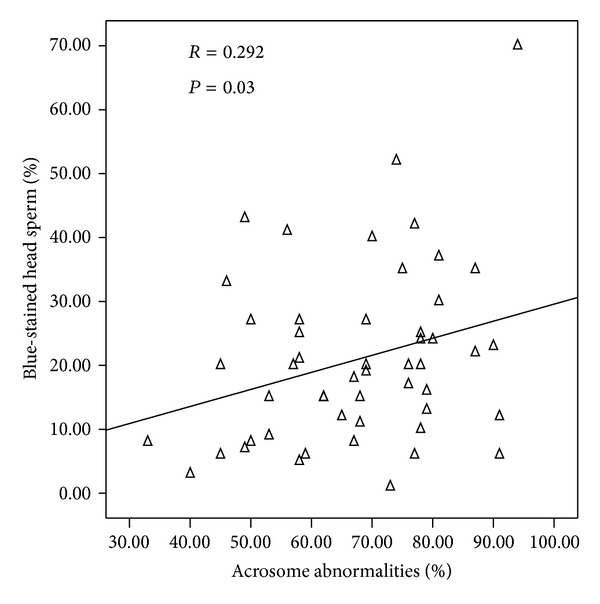
Correlation between sperm nucleus maturity and acrosome abnormalities.

The rate of spermatozoa with blue-stained nuclei was negatively correlated with the percentage of those with normal morphology (Figure 2). We also found significant and positive correlations between chromatin maturity and sperm head abnormalities (Figure 3) and acrosome abnormalities (Figure 4).

**Figure 5:**
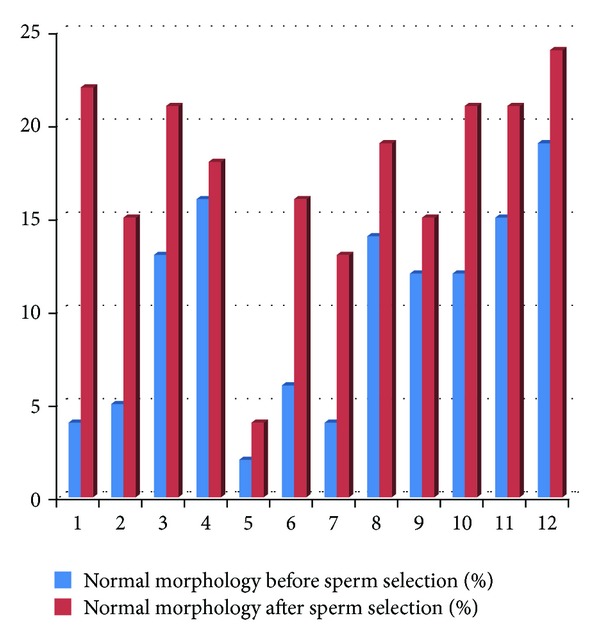
Normal morphology before and after sperm selection (*n* = 12).

**Figure 6:**
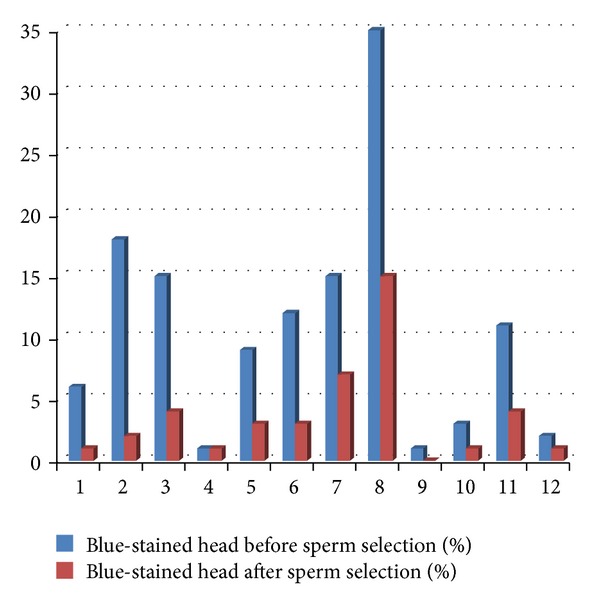
Nuclear maturity before and after sperm selection (*n* = 12).

The sperm selection by density gradient increased significantly the sperm total motility and decreased significantly the rates of sperm with abnormal morphology and immature chromatin (Table 3, Figures 5 and 6).

## 4. Discussion

According to some literature data [14, 22, 23], our study demonstrated that there is no correlation between the degree of sperm chromatin condensation assessed by AB staining and some sperm parameters, including motility, vitality, and sperm count. These results suggested that chromatin condensation constitutes a valuable parameter in the assessment of male fertility, completely independent of conventional sperm parameters [24]. In contrast, other studies reported significant correlations between sperm chromatin immaturity and the decrease of sperm count and progressive motility [17, 25]. In fact, protamination anomalies are probably related to a generalized abnormal spermiogenesis with defective semen parameters in result [25]. However, the inherent difficulties with the AB assay are the visualization and accurate counting of the mature sperm that are clear colored particularly in oligozoospermic ejaculates with reduced sperm concentration. Several different counterstains were tested, including rose bengal stain, janus green stain and eosin Y stain which gave the best results and was therefore used to improve the visualization of excessive histones in sperm [26, 27].

Furthermore, we founded a correlation between the normal sperm morphology and the nuclear maturity. We noted also that the degree of sperm chromatin compaction was significantly correlated with the average number of sperm head abnormalities and the acrosome abnormalities. These results confirm earlier investigations [12, 19, 24, 28–30] that demonstrated significant correlations between sperm morphology and its nuclear condensation. Similarly, Kazerooni et al. [17] showed a significantly higher percentage of AB-positive spermatozoa among men with teratozoospermia when compared with normozoospermic group (31.6% versus 14.1%; *P* < 0.001). More recently, Zini et al. [31] showed significant correlation between morphological sperm head defects and nuclear condensation anomalies and suggested that these sperm dysmorphisms may in part due to incomplete sperm nuclear compaction. These results were in accordance with those of Boitrelle et al. [32] who used high-magnification (×10000) contrast microscopy to study sperm morphology and demonstrated that the presence of sperm head vacuoles was associated with a complete or partial failure of chromatin condensation. However, Adham et al. [33] reported that the morphological changes of the acrosomal vesicle during spermatogenesis are concomitant with chromatin condensation. Therefore, it could be suggested that the failure of the initial chromatin condensation during spermatogenesis leads to impairment of acrosome attachment to the nucleus involvement or to dehiscence of acrosome from the nucleus [33]. In the opposite, no correlation was found between sperm morphology and sperm condensation evaluated by AB test in other studies [14].

In light of these results, we suggest that some alterations in the process of spermiogenesis may promote multiple defects in remodeling and compaction of the sperm chromatin. On the basis of some recent studies [8, 34], it has been shown that protamine P2 precursors play a pivotal role in maintaining the P1/P2 ratio. Thus, any defect in pre-P2 mRNA translation appears to cause abnormal sperm morphogenesis, reduced sperm motility, and subsequent male infertility [35, 36].

Secondly, the compaction of the nuclear chromatin and the DNA integrity are two linked parameters [10, 37–39]. To explore simultaneously these important criteria, acridine orange staining was used concomitantly to the AB test [17, 40], and a significant correlation was found between the two dyes. Thereby, the nuclei protamination requires a spatial change in DNA structure with the intervention of endogenous nucleases such as topoisomerase II which plays an important role in the phenomena of breakage and repair sperm DNA [41, 42]. So when repair systems are not sufficiently effective, the nuclear DNA fragmentation may increase [43].

Similarly, many studies reported an association between sperm nuclei maturity evaluated by AB test and some DNA abnormalities in ejaculated human sperm [32, 44–46]. Indeed, the common factor underlying sperm immaturity and aneuploidies is the diminished expression of HspA2 whose lack may cause diminished cellular transport of proteins, such as DNA-repair enzymes essential during spermiogenesis [47]. Kovanci et al. [47] demonstrated a close correlation between the incidence of immature spermatozoa and disomies (*r* = 0.7, *P* < 0.001). These results suggested the presence of a link between chromosomal meiotic segregation and the dynamic process of nucleoproteins during male gametogenesis [48].

Currently, sperm selection techniques during medical assisted procreation are designed to obtain sperm with “ideal” motility and morphology. Otherwise, fertilization and pregnancy rate might be affected by sperm nucleus subtle anomalies that remain undetected by the biologist during the routine selection procedure [49]. For this, we used the AB staining test to evaluate the status of sperm nuclear maturity after selection, and we compared rates of sperm with immature chromatin before and after gradient density selection. We noted a significant improvement of sperm chromatin maturity after selection concomitantly with the increase of the sperm motility and the decrease of the abnormal sperm morphology rates. Our results are similar to those reported by Sanchez et al. [50] and le Lannou and Blanchard [51] studies which showed that sperm selection techniques, like swim-up migration or density gradient centrifugation, increase the proportion of sperm with normal chromatin structure. Sperm populations prepared by these techniques contained more homogeneous subpopulations, with a higher degree of nuclear maturity, but the density gradient technique would be the best procedure [51, 52]. These results are in accordance with those of Sakkas et al. [53] who indicated that both PureSperm and Percoll density gradient assays can enrich the sperm population by separating those with poorly condensed chromatin. It seems that the sperm glass-wool filtration method increases significantly the percentage of normal chromatin condensed spermatozoa in comparison with traditional swim-up or gradient density [52, 54]. 

In several previous reports, the AB tingibility of spermatozoa was indicated to correlate well with infertility [2, 20]. Talebi et al. [55] reported significant lower rates of mature spermatozoa in the group of patients with unexplained recurrent spontaneous abortion. Indeed, the clinical significance of the assessment of sperm chromatin maturity lies in its association not only with natural conception rates but also with assisted reproduction success rates [10, 56]. Sadeghi et al. [16] believed that  the fertilization rate following intracytoplasmic sperm injection (ICSI) was significantly increased in the group with less than 10% abnormal chromatin using AB test. These results were in agreement with the study of Esterhuizen et al. [57] who showed that fertilization rate in ICSI is lower when semen samples with high rates of nuclear condensation are used. Other studies reported that sperm with high rates of blue-stained nucleus can affect fertilization rate following *in vitro* fertilization procedure and pregnancy rates [58, 59]. Also, the risk of spontaneous abortion would be significantly higher after artificial insemination using ejaculates with high rates of immature nuclear sperm [60, 61].

So, it has been shown that a sperm with protamine deficiency and increased histone remnants leads to premature chromatin condensation that is the cause of failures in fertilization and embryo development [1, 62]. Moreover, the chromatin of sperm with reduced amounts of protamines was observed to be susceptible to chemical disruption [63].

 In contrast, Hammadeh et al. [64] found no difference in fertilization rate in patients with ejaculated sperms or sperms obtained from testicular extracts, although there was a significant difference in the percentage of AB staining between the two groups (ejaculated spermatozoa 33.1 ± 18.9 and spermatozoa extracted from testis biopsy 70.0 ± 17.7 with *P* < 0.0001). However, unlike aniline blue staining, they found a significant difference in the percentage of CMA3-positive sperm between the two groups. This difference between AB and CMA3 staining could be due to the fact that CMA3 staining is a more sensitive and specific test and CMA3 reveals not only the presence of excessive histones but also protamine deficiency [59]. Similarly, Razavi et al. [49] did not find any correlation between excessive histones evaluated by AB staining and fertilization rate following ICSI. This could be explained by the presence of confounding factors such as ICSI procedure operator's selection of spermatozoa according to normal morphology that may influence the effect of sperm chromatin status on ICSI outcomes [16].

## 5. Conclusions

To assess the sperm nuclear maturity in the exploration of male infertility we can use simple techniques, such as AB staining, in addition to classic sperm exploration methods as spermogram and sperm selection assays. Our study provides additional data on the correlation between the quality of sperm nuclear material and morphological defects, primarily the sperm head anomalies. Sperm chromatin integrity is essential for successful fertilization, embryo development, and normal pregnancy, and a protamine deficiency appeared to effect fertilization rate and embryo quality. Therefore, there are still controversies over the effect of sperm chromatin status on fertilization rate, embryo quality, and pregnancy outcomes. The use of more sophisticated techniques as chromomycin A3 assay (CAM3) [59] and transmission electron microscopy (TEM) image cytometry [65] provides more accurate and specific results in the evaluation of the sperm nucleus maturity but has many technical constraints to use in routine investigations. We must continue and deepen the study of sperm chromatin quality in infertile men to better understand their relationship with the fertilizing capacity and better assess their impact on the results of *in vitro* fertilization and embryo development.

## Figures and Tables

**Table 1:** Means and ranges of semen parameters and AB tingibility of sperm nuclear in the study samples (*n* = 51).

	Mean ± SD	Ranges
Volume (mL)	3.42 ± 1.73	0.9–11
Total motility (%)	40.78 ± 11.15	15–55
Rapid progressive motility “a” (%)	13.72 ± 7.60	0–25
Slowly progressive motility “b” (%)	21.37 ± 6.08	5–35
No progressive motility “c” (%)	5.78 ± 2.09	5–15
Sperm concentration (millions/mL)	75.20 ± 64.05	6.20–392
Vitality (%)	76.19 ± 10.32	45–97
Normal morphology (%)	9.09 ± 6.97	1–30
Unstained (mature) sperm head (%)	79.45 ± 13.44	30–99
Blue-stained (immature) sperm head (%)	20.66 ± 13.76	1–70

**Table 2:** Comparison of semen parameters between G1 and G2 groups.

Spermogram data (Mean ± SE)	G1 (*n* = 31)	G2 (*n* = 20)	*P* value
Volume (mL)	3.43 ± 1.96	3.41 ± 1.40	0.97
Total motility (%)	42 ± 12	39.04 ± 9.8	0.34
Rapid progressive motility “a” (%)	14.1 ± 8.25	12.14 ± 6.43	0.19
Slowly progressive motility “b” (%)	21.33 ± 6.28	21.42 ± 5.94	0.95
No progressive motility “c” (%)	5.83 ± 2.30	5.71 ± 1.79	0.83
Sperm concentration (millions/mL)	85.58 ± 72.06	60.37 ± 48.31	0.14
Vitality (%)	76.93 ± 10.48	75.14 ± 10.25	0.54
Normal morphology (%)	10.46 ± 7.15	7.14 ± 6.36	0.08
Abnormalities of sperm head (/100 spz analyzed)	155.35 ± 32.18	176.89 ± 28.01	**0.01**
Microcephalic spz (%)	8.4 ± 5.6	13 ± 7.7	**0.02**
Acrosome abnormalities (%)	64.56 ± 14.34	71.23 ± 14.36	0.09
Cytoplasmic droplets (%)	8.20 ± 6.03	10.47 ± 8.08	0.2

**Table 3:** Comparison between total motility, rates of normal morphology, and AB tingibility of sperm nuclear before and after sperm selection (*n* = 12).

	Before sperm selection	After sperm selection	*P* value
Total motility (%)	38.24 ± 9.06	57.12 ± 10.17	**<0.001**
Normal morphology (%)	10.16 ± 5.65	17.41 ± 5.38	**0.02**
Blue-stained head sperm (%)	10.66 ± 9.67	3.50 ± 4.10	**0.01**

## References

[1] Sakkas D, Urner F, Bizzaro D (1998). Sperm nuclear DNA damage and altered chromatin structure: effect on fertilization and embryo development. *Human Reproduction*.

[2] Hammadeh ME, Stieber M, Haidl G, Schmidt W (1998). Association between sperm cell chromatin condensation, morphology based on strict criteria, and fertilization, cleavage and pregnancy rates in an IVF program. *Andrologia*.

[3] Esterhuizen AD, Franken DR, Lourens JGH, Prinsloo E, Van Rooyen LH (2000). Sperm chromatin packaging as an indicator of in-vitro fertilization rates. *Human Reproduction*.

[4] Roux C, Tripogney C, Joanne C (2004). Sperm chromatin packaging as an indicator of in-vitro fertilization rates. *Gynécologie Obstétrique et Fertilité*.

[5] Dadoune J-P (2003). Expression of mammalian spermatozoal nucleoproteins. *Microscopy Research and Technique*.

[6] Dadoune JP (1995). The nuclear status of human sperm cells. *Micron*.

[7] Hekmatdoost A, Lakpour N, Sadeghi MR (2009). Sperm chromatin integrity: etiologies and mechanisms of abnormality, assays, clinical importance, preventing and repairing damage. *Avicenna Journal of Medical Biotechnology*.

[8] Carrell DT, Emery BR, Hammoud S (2008). The aetiology of sperm protamine abnormalities and their potential impact on the sperm epigenome. *International Journal of Andrology*.

[9] Hou JW, Chen D, Jeyendran RS (1995). Sperm nuclear maturity in spinal cord-injured men: evaluation by acidic aniline blue stain. *Archives of Physical Medicine and Rehabilitation*.

[10] Agarwal A, Said TM (2003). Role of sperm chromatin abnormalities and DNA damage in male infertility. *Human Reproduction Update*.

[11] Auger J, Mesbah M, Huber C, Dadoune JP (1990). Aniline blue staining as a marker of sperm chromatin defects associated with different semen characteristics discriminates between proven fertile and suspected infertile men. *International Journal of Andrology*.

[12] Dadoune JP, Mayaux MJ, Guihard-Moscato ML (1988). Correlation between defects in chromatin condensation of human spermatozoa stained by aniline blue and semen characteristics. *Andrologia*.

[13] Hingst O, Blottner S, Franz C (1995). Chromatin condensation in cat spermatozoa during epididymal transit as studied by aniline blue and acridine orange staining. *Andrologia*.

[14] Hammadeh ME, Zeginiadov T, Rosenbaum P, Georg T, Schmidt W, Strehler E (2001). Predictive value of sperm chromatin condensation (aniline blue staining) in the assessment of male fertility. *Archives of Andrology*.

[15] Talebi AR, Moein MR, Tabibnejad N, Ghasemzadeh J (2008). Effect of varicocele on chromatin condensation and DNA integrity of ejaculated spermatozoa using cytochemical tests. *Andrologia*.

[16] Sadeghi MR, Hodjat M, Lakpour N (2009). Effects of sperm chromatin integrity on fertilization rate and embryo quality following intracytoplasmic sperm injection. *Avicenna Journal of Medical Biotechnology*.

[17] Kazerooni T, Asadi N, Jadid L (2009). Evaluation of sperm’s chromatin quality with acridine orange test, chromomycin A3 and aniline blue staining in couples with unexplained recurrent abortion. *Journal of Assisted Reproduction and Genetics*.

[18] Andreetta AM, Stockert JC, Barrera C (1995). A simple method to detect sperm chromatin abnormalities: cytochemical mechanism and possible value in predicting semen quality in assisted reproductive procedures. *International Journal of Andrology*.

[19] Hofmann N, Hilscher B (1991). Use of aniline blue to assess chromatin condensation in morphologically normal spermatozoa in normal and infertile men. *Human Reproduction*.

[20] Foresta C, Zorzi M, Rossato M, Varotto A (1992). Sperm nuclear instability and staining with aniline blue: abnormal persistance of histones in spermatozoa in infertile men. *International Journal of Andrology*.

[21] WHO World Health Organization (1999). *Laboratory Manual for the Examination of Human Semen and Sperm-Cervical Mucus Interaction*.

[22] Salsabili N, Mehrsai A, Jalalizadeh B (2006). Correlation of sperm nuclear chromatin condensation staining method with semen parameters and sperm functional tests in patients with spinal cord injury, varicocele, and idiopathic infertility. *Urology Journal*.

[23] Sadek A, Almohamdy ASA, Zaki A, Aref M, Ibrahim SM, Mostafa T (2011). Sperm chromatin condensation in infertile men with varicocele before and after surgical repair. *Fertility and Sterility*.

[24] Kim HS, Kang MJ, Kim SA (2013). The utility of sperm DNA damage assay using toluidine blue and aniline blue staining in routine semen analysis. *Clinical and Experimental Reproductive Medicine*.

[25] Aoki VW, Liu L, Carrell DT (2005). Identification and evaluation of a novel sperm protamine abnormality in a population of infertile males. *Human Reproduction*.

[26] Wong A, Chuan SS, Patton WC, Jacobson JD, Corselli J, Chan PJ (2008). Addition of eosin to the aniline blue assay to enhance detection of immature sperm histones. *Fertility and Sterility*.

[27] Park YS, Kim MK, Lee SH (2011). Efficacy of testicular sperm chromatin condensation assay using aniline blue-eosin staining in the IVF-ET cycle. *Clinical and Experimental Reproductive Medicine*.

[28] Leiva S, Loyola M, Agar AM, Bustos-Obregón E (1994). Evaluation of DNA/protein status and nuclear maturity of human sperm. *Cytobios*.

[29] Franken DR, Franken CJ, de la Guerre H, de Villiers A (1999). Normal sperm morphology and chromatin packaging: comparison between aniline blue and chromomycin A3 staining. *Andrologia*.

[30] Lazaros LA, Vartholomatos GA, Hatzi EG (2011). Assessment of sperm chromatin condensation and ploidy status using flow cytometry correlates to fertilization, embryo quality and pregnancy following in vitro fertilization. *Journal of Assisted Reproduction and Genetics*.

[31] Zini A, Phillips S, Courchesne A (2009). Sperm head morphology is related to high deoxyribonucleic acid stainability assessed by sperm chromatin structure assay. *Fertility and Sterility*.

[32] Boitrelle F, Ferfouri F, Petit JM (2011). Large human sperm vacuoles observed in motile spermatozoa under high magnification: nuclear thumbprints linked to failure of chromatin condensation. *Human Reproduction*.

[33] Adham IM, Nayernia K, Burkhardt-Göttges E (2001). Teratozoospermia in mice lacking the transition protein 2 (Tnp2). *Molecular Human Reproduction*.

[34] Torregrosa N, Domínguez-Fandos D, Camejo MI (2006). Protamine 2 precursors, protamine 1/protamine 2 ratio, DNA integrity and other sperm parameters in infertile patients. *Human Reproduction*.

[35] Tseden K, Topaloglu O, Meinhardt A (2007). Premature translation of transition protein 2 mRNA causes sperm abnormalities and male infertility. *Molecular Reproduction and Development*.

[36] Nanassy L, Liu L, Griffin J, Carrell DT (2011). The clinical utility of the protamine 1/protamine 2 ratio in sperm. *Protein and Peptide Letters*.

[37] de Iuliis GN, Thomson LK, Mitchell LA (2009). DNA damage in human spermatozoa is highly correlated with the efficiency of chromatin remodeling and the formation of 8-hydroxy-2′-deoxyguanosine, a marker of oxidative stress. *Biology of Reproduction*.

[38] Schulte RT, Ohl DA, Sigman M, Smith GD (2010). Sperm DNA damage in male infertility: etiologies, assays, and outcomes. *Journal of Assisted Reproduction and Genetics*.

[39] Aitken RJ, De Iuliis GN (2009). On the possible origins of DNA damage in human spermatozoa. *Molecular Human Reproduction*.

[40] Erenpreiss J, Bars J, Lipatnikova V, Erenpreisa J, Zalkalns J (2001). Comparative study of cytochemical tests for sperm chromatin integrity. *Journal of Andrology*.

[41] Sakkas D, Mariethoz E, Manicardi G, Bizzaro D, Bianchi PG, Bianchi U (1999). Origin of DNA damage in ejaculated human spermatozoa. *Reviews of Reproduction*.

[42] Henkel R, Hajimohammad M, Stalf T (2004). Influence of deoxyribonucleic acid damage on fertilization and pregnancy. *Fertility and Sterility*.

[43] Sakkas D, Manicardi G, Bianchi PG, Bizzaro D, Bianchi U (1995). Relationship between the presence of endogenous nicks and sperm chromatin packaging in maturing and fertilizing mouse spermatozoa. *Biology of Reproduction*.

[44] Morel F, Mercier S, Roux C, Elmrini T, Clavequin MC, Bresson JL (1998). Interindividual variations in the disomy frequencies of human spermatozoa and their correlation with nuclear maturity as evaluated aniline blue staining. *Fertility and Sterility*.

[45] Sati L, Ovari L, Bennett D, Simon SD, Demir R, Huszar G (2008). Double probing of human spermatozoa for persistent histones, surplus cytoplasm, apoptosis and DNA fragmentation. *Reproductive BioMedicine Online*.

[46] Óvári L, Sati L, Stronk J, Borsos A, Ward DC, Huszar G (2010). Double probing individual human spermatozoa: aniline blue staining for persistent histones and fluorescence in situ hybridization for aneuploidies. *Fertility and Sterility*.

[47] Kovanci E, Kovacs T, Moretti E (2001). FISH assessment of aneuploidy frequencies in mature and immature human spermatozoa classified by the absence or presence of cytoplasmic retention. *Human Reproduction*.

[48] Morel F, Roux C, Bresson JL (2001). Disomy frequency estimated by multicolour fluorescence in situ hybridization, degree of nuclear maturity and teratozoospermia in human spermatozoa. *Reproduction*.

[49] Razavi S, Nasr-Esfahani MH, Mardani M, Mafi A, Moghdam A (2003). Effect of human sperm chromatin anomalies on fertilization outcome post-ICSI. *Andrologia*.

[50] Sanchez R, Villagran E, Risopatron J, Celis R (1994). Evaluation of nuclear maturity in human spermatozoa obtained by sperm-preparation methods. *Andrologia*.

[51] le Lannou D, Blanchard Y (1988). Nuclear maturity and morphology of human spermatozoa selected by Percoll density gradient centrifugation or swim-up procedure. *Journal of Reproduction and Fertility*.

[52] Hammadeh ME, Kühnen A, Amer AS, Rosenbaum P, Schmidt W (2001). Comparison of sperm preparation methods: effect on chromatin and morphology recovery rates and their consequences on the clinical outcome after in vitro fertilization embryo transfer. *International Journal of Andrology*.

[53] Sakkas D, Manicardi GC, Tomlinson M (2000). The use of two density gradient centrifugation techniques and the swim-up method to separate spermatozoa with chromatin and nuclear DNA anomalies. *Human Reproduction*.

[54] Henkel RR, Franken DR, Lombard CJ, Schill W-B (1995). Selective capacity of glass-wool filtration for the separation of human spermatozoa with condensed chromatin: a possible therapeutic modality for male-factor cases?. *Journal of Assisted Reproduction and Genetics*.

[55] Talebi AR, Vahidi S, Aflatoonian A (2012). Cytochemical evaluation of sperm chromatin and DNA integrity in couples with unexplained recurrent spontaneous abortions. *Andrologia*.

[56] Jeulin C, Feneux D, Serres C (1986). Sperm factors related to failure of human in-vitro fertilization. *Journal of Reproduction and Fertility*.

[57] Esterhuizen AD, Franken DR, Lourens JGH, van Zyl C, Müller II, Van Rooyen LH (2000). Chromatin packaging as an indicator of human sperm dysfunction. *Journal of Assisted Reproduction and Genetics*.

[58] Liu DY, Baker HWG (1992). Sperm nuclear chromatin normality: relationship with sperm morphology, sperm-zona pellucida binding, and fertilization rates in vitro. *Fertility and Sterility*.

[59] Nasr-Esfahani MH, Razavi S, Mardani M (2001). Relation between different human sperm nuclear maturity tests and in vitro fertilization. *Journal of Assisted Reproduction and Genetics*.

[60] Mohamed EE, Mohamed MA (2012). Effect of sperm chromatin condensation on the outcome of intrauterine insemination in patients with male factor infertility. *Journal of Reproductive Medicine*.

[61] Lin M-H, Kuo-Kuang Lee R, Li S-H, Lu C-H, Sun F-J, Hwu Y-M (2008). Sperm chromatin structure assay parameters are not related to fertilization rates, embryo quality, and pregnancy rates in in vitro fertilization and intracytoplasmic sperm injection, but might be related to spontaneous abortion rates. *Fertility and Sterility*.

[62] Oliva R (2006). Protamines and male infertility. *Human Reproduction Update*.

[63] Cho C, Willis WD, Goulding EH (2001). Haploinsufficiency of protamine-1 or -2 causes infertility in mice. *Nature Genetics*.

[64] Hammadeh ME, Al-Hasani S, Doerr S (1999). Comparison between chromatin condensation and morphology from testis biopsy extracted and ejaculated spermatozoa and their relationship to ICSI outcome. *Human Reproduction*.

[65] Auger J, Schoevaert D, Negulesco I, Dadoune J (1993). The nuclear status of human sperm cells by TEM image cytometry: nuclear shape and chromatin texture in semen samples from fertile and infertile men. *Journal of Andrology*.

